# Fundamentals of diagnostic ultrasonography in sheep and goat medicine: a comprehensive illustrated overview

**DOI:** 10.3389/fvets.2025.1562097

**Published:** 2025-03-17

**Authors:** Mohamed Marzok, Mohamed Tharwat

**Affiliations:** ^1^Department of Clinical Sciences, College of Veterinary Medicine, King Faisal University, Al-Ahsa, Saudi Arabia; ^2^Department of Clinical Sciences, College of Veterinary Medicine, Qassim University, Buraidah, Saudi Arabia

**Keywords:** animals, goat, sheep, small ruminants, ultrasonography

## Abstract

This illustrated review emphasizes the fundamentals of diagnostic ultrasonography in sheep and goat medicine. The procedure can effectively assess the thoracic and abdominal organs in both healthy and diseased states. The review discusses five main sections. The first one clarifies the principles of pulmonary sonography in sheep and goats followed by image finding in animals with respiratory disorders including pneumonia, pleuropneumonia, lung abscessation and pleuritis. Second section shows the fundamentals of echography in sheep and goats followed by scanning of animals with cardiovascular disorders including heart failure, fibrinous pericarditis, endocarditis and nutritional muscular dystrophy. Third section of this review discusses the principles of gastrointestinal sonography in sheep and goats followed by picture in some digestive disorders including peritonitis, enteritis and retroperitoneal abscessation. Fourth part shows the basics in hepatic and biliary tissue followed by sonography of sheep and goats with hepatic and biliary disorders including fatty liver, hepatitis cysticercosis, cholangitis, cholecystitis, cholestasis and choledocholithiasis. Last section of this review discusses the fundamentals of urinary system ultrasonography followed by imaging of the urinary disorders including renal failure, hydronephrosis, pyelonephritis, obstructive urolithiasis, cystitis and paralysis of the urinary bladder. In conclusion, ultrasonography of either healthy or diseased sheep or goats is very useful for assessing the normal structure and function of both healthy and dysfunctional organs. It is highly recommended to adopt this procedure as a standard preliminary method for examining sheep and goats with any medical condition.

## 1 Introduction

Sheep and goats are vital sources of food, fiber, and income for rural populations, but they are often managed under low-input systems, making them susceptible to health issues (1). Ultrasonography has become an essential tool in veterinary practice, particularly for small ruminants as it is non-invasive and real-time imaging tool that allows for detailed internal assessments without the need for surgery (2). Given the small size of ruminants like sheep and goats, ultrasonography provides an efficient way to detect abnormalities, monitor pregnancies, and diagnose various medical conditions with minimal stress to the animals (2). This non-invasive imaging technique uses the transmission and reception of sound waves to create two-dimensional grey-scale images of the internal structural anatomy. From its early use as an experimental technique in the late 1950s and early 1960s, basic ultrasonography has now become standard for most domestic species in veterinary practice, with various individual applications.

Ultrasonography is a safe and effective method providing excellent and detailed images of organ morphology and pathology. It is a non-invasive technique that can be used to evaluate the health of internal organs without the stress of an invasive surgical approach. Ultrasonography is now the only practical method of non-invasive imaging for many of the most important pathological processes in small ruminants. It has the potential to significantly improve productivity and welfare by enabling early detection of pathologies that often go undiagnosed, thereby minimizing adverse events following treatment (3).

In our clinic, we have used ultrasonography for diagnosing sheep and goats with respiratory (4), digestive (5), hepatic (6–8) and urinary (3, 9, 10) disorders. This review article was designed to investigate the principles of diagnostic ultrasonography in sheep and goat with thoracic and abdominal disorders. The procedure can also be used to evaluate the thoracic and abdominal organs in healthy sheep and goats.

## 2 Diseases and disorders of the lungs and pleurae

In sheep and goats, the lungs are anatomically divided into bilateral cranial lobes, each containing a cranial and caudal segment. The right lung also features a middle (cardiac) lobe and an accessory lobe that extends ventrally along the midline. Both lungs have bilateral caudal (diaphragmatic) lobes (11). Because of their grazing habits and the requirement for effective oxygen exchange in open pastures, sheep’s lungs are comparatively larger and have a more lobulated structure with finer divisions than goats’. The lungs of both animals look like spongy soft organ, surrounded the heart which was located in the mediastinum and enveloped by visceral and parietal pleura. Both animals’ lungs have an anterior apex and a posterior base. The lungs’ right and left lobes are separated into cranial and caudal parts, whereas the goat’s lung is undivided. The left lung of both animals is made up of apical and caudal lobes, with the apical being divided into cranial and caudal parts part (12).

From a histological perspective, the alveoli are approximately spherical structures that connect to alveolar ducts, alveolar sacs, or respiratory bronchioles. They consist of two primary cell types: Type-I and Type-II pneumocytes. Type-I pneumocytes form the main epithelial lining of the alveoli, characterized by their squamous shape, prominent perinuclear region, and centrally positioned nucleus. In contrast, Type-II pneumocytes are cuboidal with a central nucleus and are occasionally interspersed among the Type-I cells within the alveolar epithelium (13).

### 2.1 Ultrasonographic examination of the lungs

**Figure 1:**
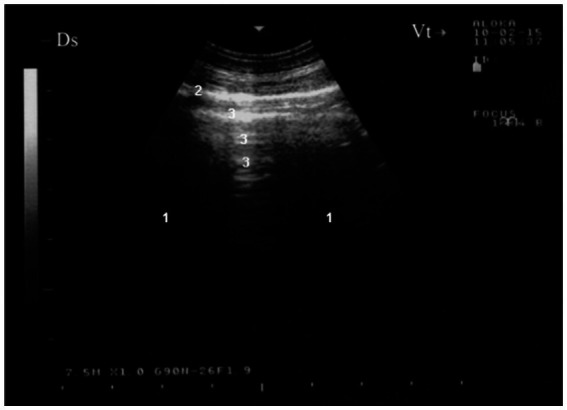
Ultrasonogram of the surface of a normal lung (7.5 MHz) in a healthy goat. Normally aerated lung parenchyma (1) does not permit penetration of the ultrasound beam, so a bright linear echo (2) and reverberation artifacts (3) are all that are seen during examination of a normal animal. Ds, dorsal; Vt, ventral (16).

As reported, ultrasonographic assessment of the dorsal lung field is preferred to start at the 9th or 10th intercostal space (14, 15). This technique is effective for identifying pleural fluid accumulation, pleural abscesses, tumors, and lung consolidation resulting from bacterial or interstitial viral pneumonia. In healthy, aerated lungs, the ultrasound beam cannot penetrate the lung tissue, producing only a bright linear echo and reverberation artifacts during the examination (Figure 1) (15–18). To best localize pulmonary lesions, each lung area may be divided in four quadrants by two lines: one horizontal parallel to the floor, passing through the shoulder joint, and one vertical passing through the 5th intercostal space. So, eight quadrants per animal are obtained and labeled alphabetically on the right side from A to D, on the left from E to H. So, each side had craniodorsal (A-E), caudodorsal (B-F), cranioventral (C-G), caudoventral (D-H) quadrant (19).

### 2.2 Respiratory disorders

#### 2.2.1 Pneumonia

Pulmonary parenchyma inflammation, primarily affecting the alveoli, is often accompanied by bronchiolar inflammation and sometimes pleuritis (20). Clinically, pneumonia is characterized by symptoms such as coughing, abnormal respiratory sounds, rapid shallow breathing, and changes in respiratory depth and pattern (20). While chest auscultation is an integral part of veterinary clinical examination, its ability to detect, localize, and specify lung pathology is limited. Although clinical examination and auscultation are valuable, they are insufficient for diagnosing many specific conditions. Therefore, supplementary diagnostic methods are necessary to confirm provisional diagnoses made during clinical evaluations (11). Ultrasonography, for instance, can reveal consolidated lung parenchyma.

#### 2.2.2 Pleuropneumonia

Pleuropneumonia, particularly contagious caprine pleuropneumonia (CCPP), is a severe respiratory disease affecting goats in various countries across Africa and Asia. It is caused by *Mycoplasma capricolum* subsp. *capripneumoniae* (*Mccp*). The acute form of CCPP is characterized by unilateral sero-fibrinous pleuropneumonia accompanied by significant pleural effusion (4, 21–24). During CCPP outbreaks in mixed herds of goats and sheep, sheep can also become infected, as evidenced by the isolation of Mccp or the presence of antibodies in clinically affected individuals. Additionally, *Mccp* has been isolated from healthy sheep, suggesting their potential role as a reservoir for the disease. Recent reports confirm CCPP in wild ruminants housed in a wildlife preservation reserve in Qatar, and it has also been identified in gazelles in the United Arab Emirates (25).

In goats, contagious caprine pleuropneumonia (CCPP) presents with symptoms such as anorexia, fever, and respiratory distress, including dyspnea, rapid shallow breathing, coughing, and nasal discharge. CCPP cause major economic losses in Africa, Asia and in the Middle East (4). Affected goats often hold their heads low, exhibit frothy nasal discharge and salivation, and show reluctance to move. Death typically occurs within 2 to 10 days following the onset of clinical signs (26). The acute and subacute forms of CCPP are marked by unilateral sero-fibrinous pleuropneumonia accompanied by significant pleural effusion. Diagnosis is based on clinical and necropsy findings, which should be confirmed through laboratory testing. Due to the challenges in isolating *Mycoplasma capricolum* subsp. *capripneumoniae* (Mccp), molecular diagnostic techniques are recommended for confirmation (4, 27).

**Figure 2:**
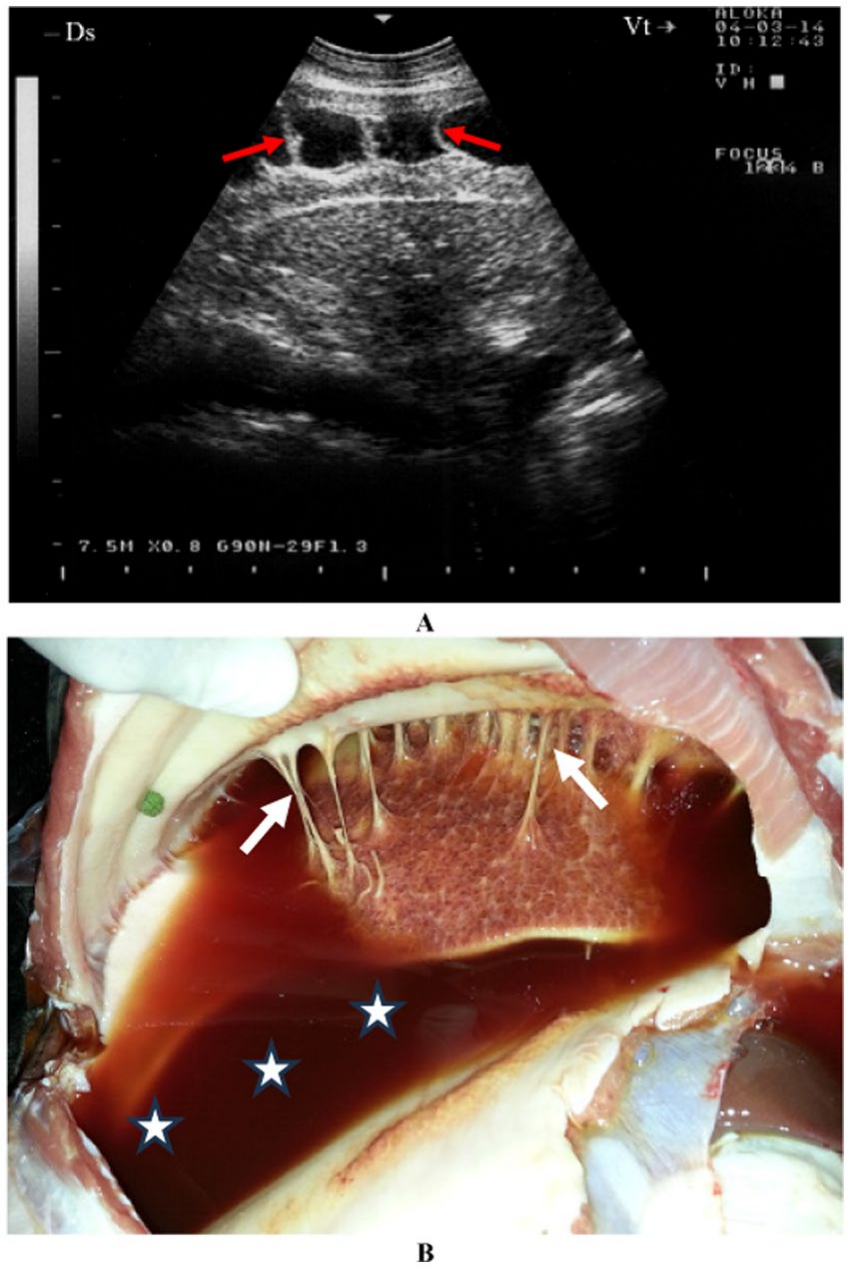
A goat with contagious caprine pleuropneumonia caused by *Mycoplasma capricolum* subsp. *Capripneumoniae.* Image **(A)** shows sonographic finding of a marked fibrinous pleurisy (red arrow). Necropsy reveals dark red pleural fluid **(B)** (stars) and fibrinous adhesions (white arrows) (4).

The presence of gas echoes within pleural or abscess fluid is a highly sensitive and specific indicator of anaerobic infection, similar to the detection of putrid breath or foul-smelling pleural fluid. Ultrasonographic evaluation of both sides of the thorax often reveals accumulations of anechoic or hypoechoic fluid in the ventral pleural space. In such cases, pleural effusion associated with pleuritis is typically unilateral due to the lack of communication between the pleural sacs. However, bilateral pleural effusion may suggest either bilateral pulmonary disease or non-inflammatory conditions such as right-sided congestive heart failure or hypoproteinemia. Pleural fluid readily transmits sound waves, appearing as an anechoic region on ultrasound. Gas pockets within pleural fluid or abscesses are visualized as bright hyperechoic spots within the anechoic area, creating a “snowstorm appearance.” The pleural fluid may be clear yellow, turbid and yellowish, reddish or dark red (4) (Figure 2).

#### 2.2.3 Chronic suppurative pneumonia (lung abscesses)

Chronic suppurative pneumonia is a common condition in sheep and goats. It can result from bacterial infections in lung tissue compromised by viral infections, inhalation of infectious agents from the oropharynx—commonly associated with *Fusobacterium necrophorum* in young lambs and kids—or hematogenous spread from septic foci in other parts of the body, such as the udder, uterus, or cellulitis lesions (28, 29).

Clinically, affected animals present with chronic weight loss, lethargy, depression, rapid breathing, occasional coughing, and mucopurulent nasal discharge. On ultrasonographic examination, the abscess capsule is often visible in well-encapsulated lesions, which may extend to occupy an entire side of the chest. For chronic respiratory diseases, penicillin administered for 20–30 days is the treatment of choice, as *Fusobacterium necrophorum* is frequently isolated (28). Regular aspiration of pulmonary abscesses combined with penicillin treatment yields promising results.

#### 2.2.4 Pleurisy (pleuritis)

Inflammation of the parietal and visceral pleura leads to fluid accumulation in the pleural cavity, characterized by varying levels of toxemia, painful shallow breathing, pleural friction sounds, and dullness on thoracic percussion due to pleural effusion (28). On ultrasonographic examination, pleural fluid is visible as hypoechoic to anechoic fluid located between the parietal pleura, diaphragm, and lung. Transudative fluid appears uniformly anechoic to hypoechoic, while fibrin appears as thin, filamentous strands floating within the effusion, loosely attached to pleural surfaces. Serosanguineous, hemorrhagic, or purulent fluid exhibits higher echogenicity compared to transudates. In fibrinous pleuritis, ultrasonography reveals separation of the pleurae and lung lobes by a hypoechoic region with acoustic enhancement of the visceral pleura. In severe cases, fibrin deposits display a hyperechoic lattice-like appearance interspersed with hypoechoic areas. Pleural abscesses present as uniform hypoechoic areas containing numerous hyperechoic spots, extending up to 16 cm in depth and often confined to one side of the thorax, with volumes of purulent material (pyothorax) reaching 2–3 liters (4, 20).

## 3 Diseases and disorders of the cardiovascular system

### 3.1 Anatomical background

In sheep and goats, the heart extends from the 3rd to the 6th rib and may make contact with the diaphragm along its caudal edge. Its position and orientation within the thorax are similar to those of other ruminants (11, 20, 28, 29). Histologically, cardiac muscle fibers appear in longitudinal sections, with visible striations along the length of the muscle fibers. The nuclei of the cardiac muscle cells are centrally located within the cells. In a well-prepared section, the nucleolus is prominently stained, and the surrounding nucleus displays a delicate pattern. The myofibrils often bypass the nucleus, and a perinuclear region, devoid of striations, can be seen. This region contains cytoplasmic organelles that are not directly involved in muscle contraction. Each muscle fiber is encased in an endomysium of delicate connective tissue, which includes a dense network of capillaries. Although the reticular fibers of the endomysium are typically not visible, the nuclei of fibroblasts, which lie between the muscle fibers and the numerous capillaries alongside the fibers, are easily seen. The fibroblast nuclei are typically more flattened and darker-stained compared to the cardiac muscle cell nuclei, and they are located peripherally (13).

### 3.2 Echocardiography

Echocardiography has become a standard non-invasive method for diagnosing cardiac conditions in various species. In sheep and goats, two-dimensional (B-mode) echocardiography is increasingly utilized to diagnose suspected heart disease (30–41).

#### 3.2.1 Right parasternal ultrasonogram

**Figure 3:**
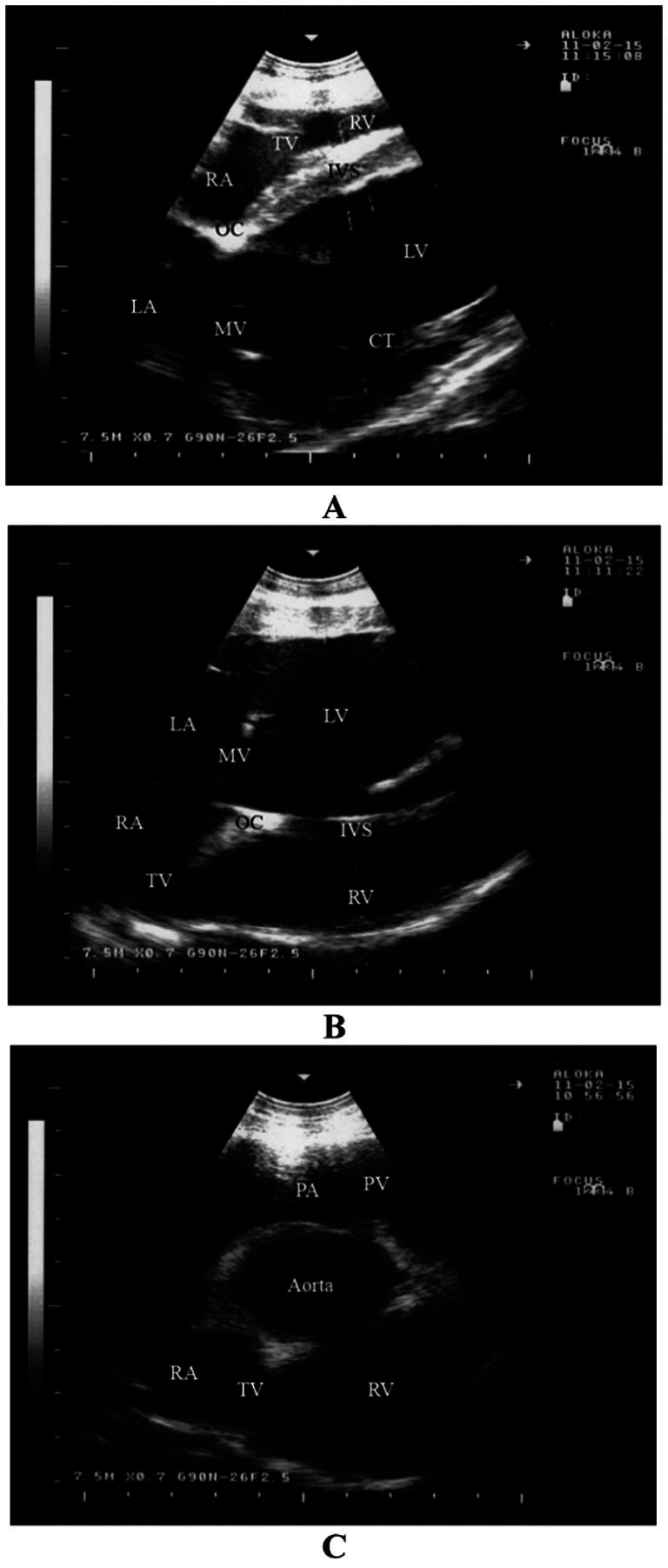
Echocardiography in a healthy sheep. Image **(A)** shows a right parasternal caudal long-axis view of the right and left ventricles (four-chamber view) obtained from the right 4th intercostal space. The chordae tendinae of the mitral valve are seen as echoic lines. Image **(B)** shows a left parasternal caudal long-axis, where the four cardiac chambers are observed as well as the atrioventricular valves. Image **(C)** shows a left parasternal cranial long-axis view of the right ventricular outflow tract where the pulmonary artery and aorta are imaged. IVS, interventricular septum; LA, left atrium; LV, left ventricle; MV, mitral valve; RA, right atrium; RV, right ventricle; TV, tricuspid valve; CT, chordae tendinae; OC, ossa chordis (16).

When the probe is positioned longitudinally in the right 4th intercostal space, the caudal long-axis four-chamber view of the ventricles, atria, and interventricular septum is imaged (Figure 3A) (16). This view provides images of the right and left ventricles, the right and left atria, the mitral and tricuspid valves, and the interventricular septum. Additionally, the ossa chordis appears as a hyperechoic area in this position (30–34, 37, 39).

#### 3.2.2 Left parasternal ultrasonogram

When the probe is positioned longitudinally in the left 4th intercostal space, it provides a view of the ventricles, atria, and atrioventricular valves. This view includes images of the left and right ventricles, the right and left atria, the mitral and tricuspid valves, and the interventricular septum (Figure 3B) (16). The ossa chordis also appears as a hyperechoic area in this position (30–34, 37, 39). The right ventricular outflow tract is visible from the 3rd intercostal space on the left side (Figure 3C) (16). In this view, the right ventricle, tricuspid valve, and right atrium are imaged, along with the pulmonary artery, pulmonary valve, aorta, and aortic valve (30–34, 37, 39, 42).

### 3.3 Cardiovascular disorders

#### 3.3.1 Heart failure

Heart failure occurs when the heart cannot pump effectively enough to meet the body’s blood flow requirements. Several condition can cause heart failure in sheep and goats such as valvular diseases, endocarditis, myocardial diseases, cardiomyopathy, core pulmonale, cardiac toxicosis and pericarditis (20, 68, 70). It can manifest as either right-sided or left-sided failure and may result from conditions affecting the heart valves (e.g., endocarditis) or the myocardium (e.g., myocarditis). Congenital heart defects, including ventricular or atrial septal defects, tetralogy of Fallot, and patent ductus arteriosus, can also lead to heart failure (11, 28, 29, 36). It is important to note that while hypoproteinemia can cause ventral and submandibular edema, it does not lead to jugular or mammary vein distention and pulsation. Therefore, key indicators of heart failure in livestock include venous distention and pulsation combined with abnormal heart sounds or rhythms (20). Ultrasonographic evaluations may reveal subcutaneous fluid accumulation (anasarca), as well as pericardial, pleural, and peritoneal effusions, and intestinal edema. Treatment options for sheep and goats are limited to stall rest and the use of diuretics administered parenterally, in addition to addressing the underlying cause, if identifiable (11, 28, 29).

#### 3.3.2 Fibrinous pericarditis

**Figure 4:**
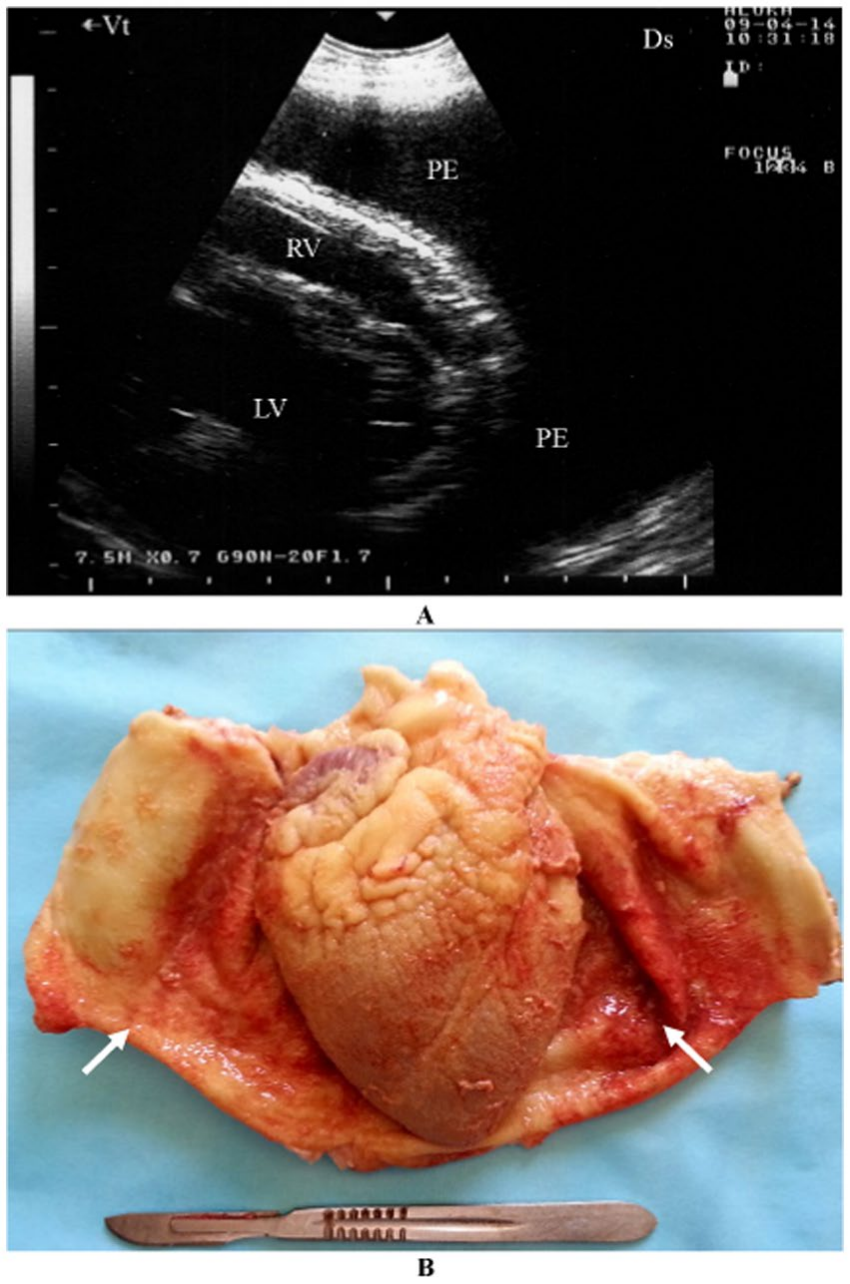
Pericardial effusions and echogenic tags of epicardium in CCPP goat **(A)**. Necropsy showed thickened and roughened surfaces of the pericardium (arrows) **(B)** (4). PE, pericardial effusion; RV, right ventricle; LV, left ventricle.

Pericarditis is an uncommon condition in sheep and goats. A female Nagde sheep was brought to the Veterinary Teaching Hospital at Qassim University with a seven-day history of reduced appetite, along with symptoms including lethargy, dyspnea, coughing, nasal discharge, and an elevated respiratory rate. Mycoplasma species, particularly *Mycoplasma capricolum subsp. capripneumoniae* and *Mycoplasma mycoides subsp. mycoides*, are often incriminated. Ultrasonographic evaluation of both sides of the thorax revealed significant pleural and pericardial effusions containing anechoic to echogenic fluid, lung parenchyma consolidation, and fibrinous pleuritis. Fibrin strands were also observed extending from the epicardium and floating in the fluid. Postmortem findings included extensive mucopurulent pleuropneumonia, serosanguineous pleural and pericardial effusions, and fibrinous pericarditis (Figure 4). Histopathological analysis showed substantial thickening of the pericardium due to fibrin deposition and edema, along with moderate infiltration of loosely arranged lymphocytes. Some regions exhibited scattered neutrophils, fragmented necrotic nuclear debris, and multiple localized areas of necrosis within the pericardium.

#### 3.3.3 Endocarditis

Compared to cattle, detailed clinical reports on ovine and caprine vegetative endocarditis are relatively scarce, primarily because the condition often occurs without an audible murmur. Bacteremia plays a crucial role in the development of bacterial endocarditis. In cattle, *Corynebacterium pyogenes* is the most frequently isolated pathogen from blood and endocardial lesions, although *Streptococcus* species, *Staphylococcus* species, and Gram-negative bacteria can also cause the disease. The tentative diagnosis of vegetative endocarditis relies on clinical signs such as depression, chronic weight loss, recumbency, pain, fever, polyarthritis, shifting weight due to discomfort, and dyspnea. Ultrasonographic examination is typically used for clinical diagnosis, which is later confirmed through postmortem examination. Attempts to treat ovine vegetative endocarditis with procaine penicillin (44,000 IU/kg daily for at least 10 days) have generally been unsuccessful (20).

#### 3.3.4 Nutritional muscular dystrophy

Nutritional muscular dystrophy, also known as white muscle disease, can be either congenital or acquired and may affect both cardiac and skeletal muscles. This condition is a leading cause of sudden death in young kids due to cardiac failure. It is the most prevalent disorder linked to selenium and/or vitamin E deficiency in sheep and goats. Inadequate levels of these nutrients hinder the body’s ability to regulate oxidative processes within cells, potentially leading to significant muscle necrosis. The cardiac muscle, diaphragm, and other skeletal muscles may be involved, resulting in diverse clinical signs that complicate accurate diagnosis (11, 28, 29). Ultrasonographic imaging of the heart typically reveals increased echogenicity in the myocardium and skeletal muscles.

## 4 Diseases and disorders of the digestive system

The caprine digestive system shares the fundamental ruminant structure and function observed in sheep, with only minor differences. However, goats possess unique anatomical and physiological traits that set them apart from sheep, reflecting their specialized feeding habits and remarkable adaptability to challenging environments unsuitable for other domesticated ruminants (11, 28, 29). The range of diseases affecting the goat digestive system is comparable to those found in sheep and cattle, with infectious and metabolic disorders being most prevalent. Although many of these diseases occur in both species, their incidence may vary. For instance, while abomasal displacement and traumatic reticulitis are common in dairy cattle, these conditions are rarely observed in goats (11, 20, 28, 29).

### 4.1 Ultrasonographic examination of the digestive system

#### 4.1.1 Rumen

**Figure 5:**
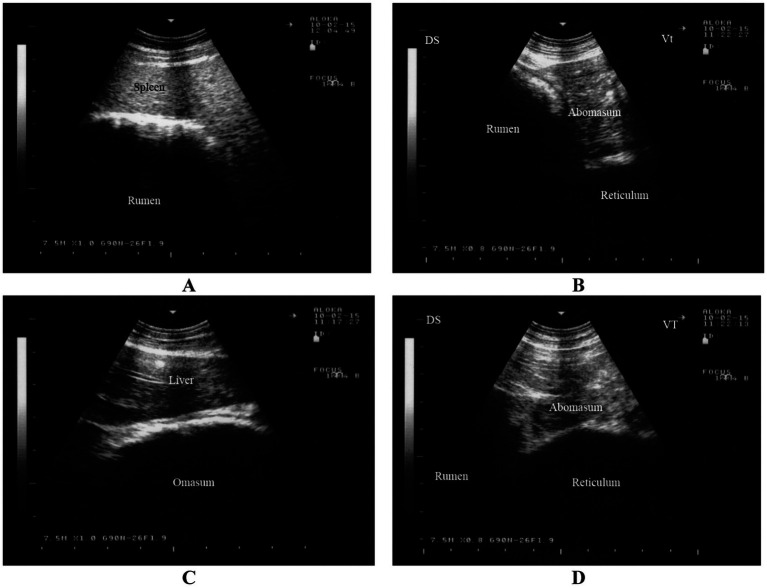
Ultrasonography of the rumen, reticulum, omasum and abomasum in a healthy goat. Image **(A)** shows an ultrasonogram of the rumen and spleen at the 11th intercostal space to the left side. Image **(B)** shows an ultrasonogram of the reticulum at the left paramedian of the sternal region. Image **(C)** shows an ultrasonogram of the omasum and liver where the omasum appears as a curved echogenic line medial to the liver. Image **(D)** shows an ultrasonogram of the abomasum caudal to the reticulum and it appears as a heterogeneous, moderately echogenic structure with multifocal echogenic foci (16).

The rumen is visible from the 9th to the 12th intercostal spaces and from the flank on the left side. Its dorsal margin runs parallel to the lung margin, extending cranioventrally to caudodorsally, while its ventral margin extends cranioventrally. The longitudinal groove of the rumen is identifiable in all goats and is located farthest from the midline of the back at the 8th intercostal space (43, 44). Positioned largely adjacent to the left abdominal wall, the rumen extends medially and appears as a thick echogenic line on imaging. Due to its gaseous contents, the internal structure of the rumen, similar to cattle, is challenging to image (Figure 5A) (16). Between the 8th and 12th intercostal spaces, the spleen is observed dorsolateral to the rumen (43, 44).

#### 4.1.2 Reticulum

The reticulum can be visualized from the *linea alba*, the left and right paramedian regions, and between the 5th and 9th intercostal spaces on both sides (44, 45). It appears as a crescent-shaped structure with a smooth contour and is located immediately adjacent to the diaphragm (Figure 5B) (16). Biphasic reticular contractions are evident, beginning with an incomplete contraction followed by partial relaxation. This is succeeded by a full contraction and subsequent complete relaxation of the organ (44, 45).

#### 4.1.3 Omasum

The omasum is visible between the 6th and 11th intercostal spaces, typically spanning 3 to 5 consecutive spaces (44, 46). Unlike in cattle, the omasum in goats is not directly adjacent to the abdominal wall but is instead separated by the liver. The organ appears as a curved echogenic line medial to the liver (Figure 5C) (16). On imaging, the omasum appears as a curved echogenic line, representing the omasal wall nearest to the transducer (44, 46).

#### 4.1.4 Abomasum

The abomasum is visualized as a heterogeneous, moderately echogenic structure with echogenic stippling, similar to its appearance in cattle. It is consistently visible from the ventral midline, as it lies directly adjacent to the abdominal wall in this region (44, 47). The abomasal folds are seen as distinct echogenic bands. The abomasum occupies a similar amount of space on both the left and right sides, extending further cranially on the left and further caudally on the right. The abomasum is seen caudal to the reticulum and appears as a heterogeneous, moderately echogenic structure with multifocal echogenic foci (Figure 5D) (16). It is important to note that the ultrasonographic length of the abomasum appears shorter than its actual length due to overlapping structures such as the reticulum and rumen (44, 47).

#### 4.1.5 Small intestine

**Figure 6:**
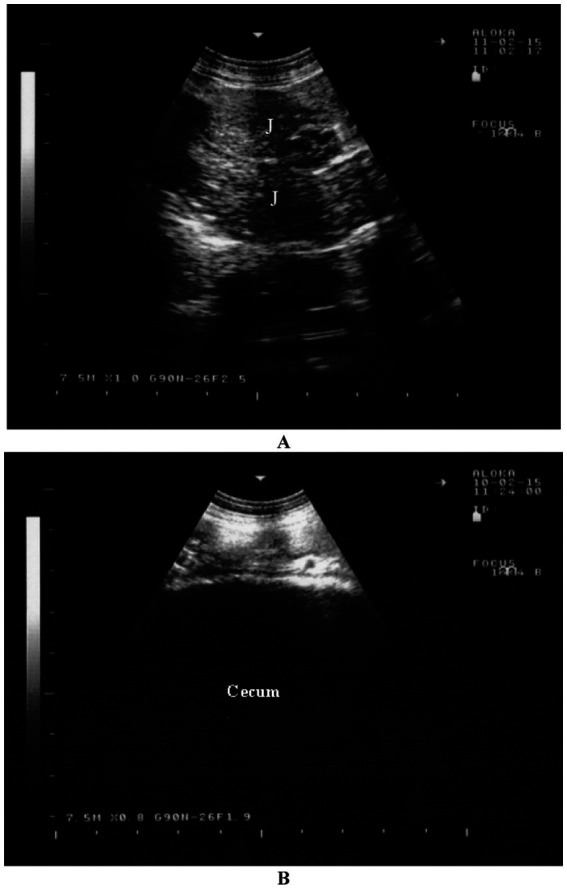
Sonographic appearance of the intestines in a healthy goat. Image **(A)** shows an ultrasonogram of the jejunum (J) in a longitudinal view in the cranioventral flank. Image **(B)** shows an ultrasonogram of the cecum located at the caudodorsal left flank (16).

The cranial part of the duodenum is typically indistinguishable from the rest of the intestinal tract, but the descending duodenum can be clearly differentiated from other segments of the small and large intestines. While the jejunum and ileum are visible, they cannot be distinguished from one another. These portions of the small intestine are most commonly observed in the ventral right flank, less often in the ventral regions of the last intercostal spaces, and occasionally in the dorsal flank. Jejunal and ileal loops are usually seen in cross-section but can also be visualized longitudinally and exhibit strong motility (Figure 6A) (16). The frequency of contractions cannot be accurately measured due to their constant motion. The intestinal contents are generally homogeneous and echogenic (44, 48).

#### 4.1.6 Large intestine

The large intestine is most frequently visible dorsally in the right flank, with occasional visibility in the ventral area. The spiral colon is easily recognized by the distinctive garland-like appearance of its centripetal and centrifugal coils. It is often positioned medially to the small intestine, not directly next to the abdominal wall. In the cecum, only the wall nearest to the transducer can be visualized (Figure 6B) (16). This wall appears as a thick, echogenic line with slight undulations (44, 48).

#### 4.1.7 Spleen

**Figure 7:**
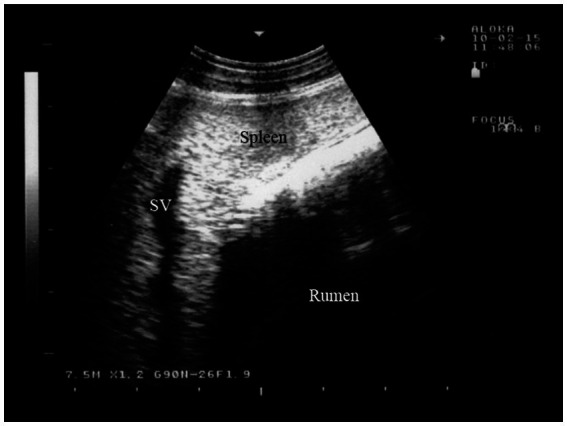
Ultrasonogram of the splenic parenchyma in a goat viewed from the 11th intercostal space to the left side. SV, splenic vein (16).

The dorsal margin of the spleen is visible running from cranioventral to caudodorsal, due to the superimposition of the lungs, similar to the liver. As a result, the distance between the dorsal visible margin of the spleen and the dorsal midline is greatest at the 8th intercostal space (ICS) and smallest just caudal to the last rib. The ventral margin follows a similar course (49). The spleen can be seen between the 8th and 12th intercostal spaces. It is located between the rumen and the abdominal wall, and in the 8th intercostal space, it is adjacent to the cranial blind sac of the rumen. The spleen is surrounded by an echogenic capsule, and its parenchyma shows a pattern of numerous weak echoes evenly distributed throughout the organ. The splenic vessels, embedded within the parenchyma, can be seen either in longitudinal or cross-section (Figure 7) (16).

### 4.2 Digestive disorders

#### 4.2.1 Peritonitis

Peritonitis is an inflammation of the peritoneum, the thin membrane that lines the inner abdominal wall and covers most of the abdominal organs. It can be localized or widespread, acute or chronic, and may result from either an infectious or non-infectious cause (67). In sheep and goats, common triggers include abdominal surgery, ruptured ulcers in the abomasum or intestines, penetration of the intestinal tract by foreign bodies, rupture of the urinary tract, rectum, uterus, or large intestine, and liver abscesses. Peritonitis may also occasionally follow conditions such as metritis, dystocia, retained fetal membranes, or pyometra. Clinically, peritonitis can be challenging to diagnose (11, 20, 50). In affected animals, ultrasonography typically shows the presence of large amounts of echogenic fluid, sometimes accompanied by fibrin tags. Postmortem examination reveals exudation and fibrin deposits within the peritoneal cavity, along with fresh adhesions that are easily broken.

#### 4.2.2 Enteritis

**Figure 8:**
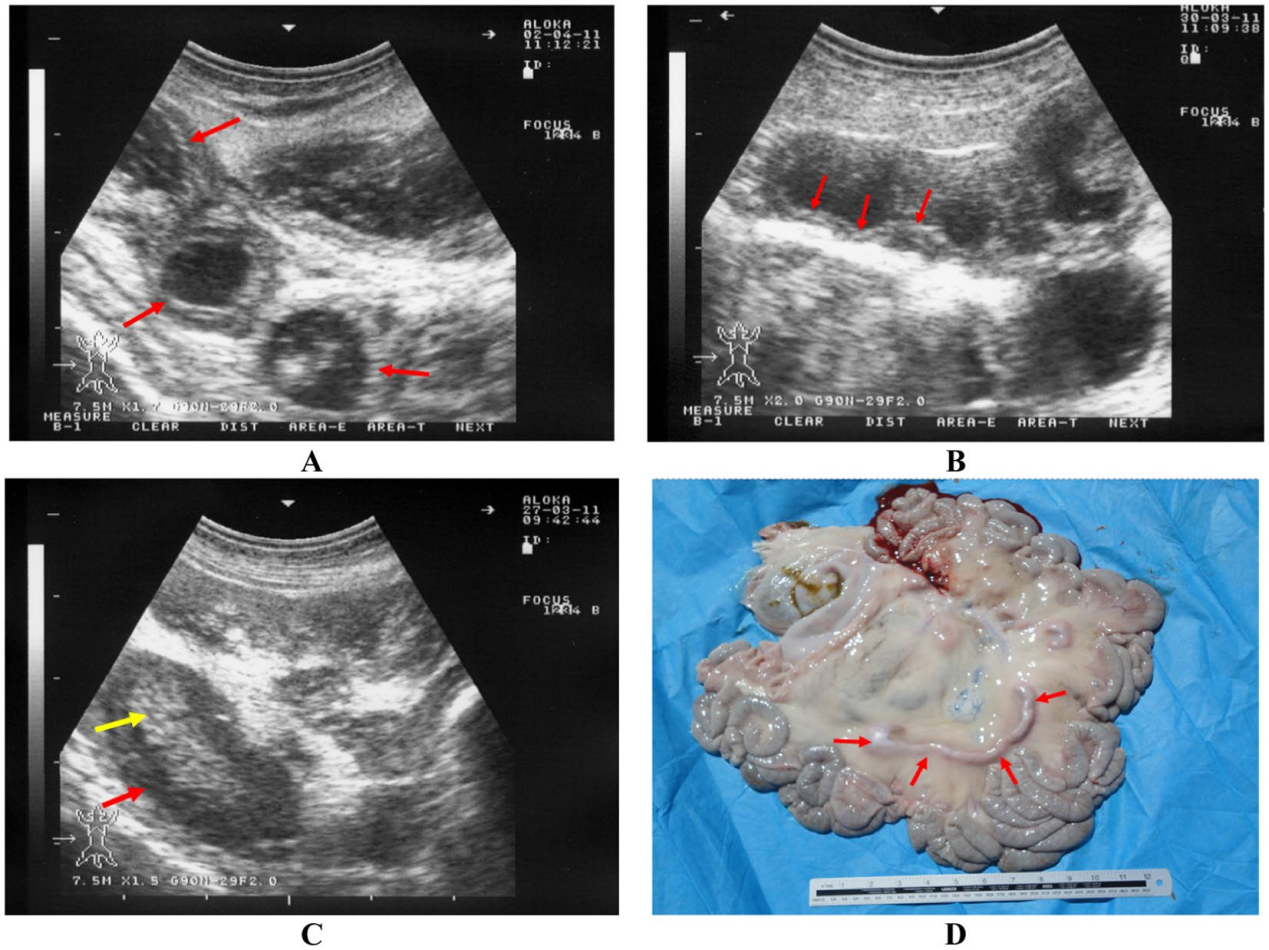
Ultrasonography and postmortem findings in a goat with paratuberculosis. Thickened intestinal walls were apparent cross sectionally **(A)** and longitudinally **(B)** (red arrows). Image **(C)** shows enlarged mesenteric lymph node with a hypoechoic cortex (red arrow) and a hyperechoic medulla (yellow arrow). Image **(D)** shows enlargement of the mesenteric lymph nodes (red arrows) (5).

Enteritis is an inflammation of the intestinal mucosa, commonly caused by factors such as abrupt dietary changes, toxicity, viral infections, parasitic infestations, and bacterial infections like salmonellosis, colibacillosis, paratuberculosis, tuberculosis, and clostridial enterotoxemia (20). Clinically, enteritis is characterized by diarrhea that may be foul-smelling, watery, and contain excessive mucus, blood, undigested food particles, and sloughed mucosal tissue. Other symptoms include dehydration, tenesmus, anorexia, colic, depression, and weight loss (11). Diagnosis is typically based on the animal’s history and clinical signs. To identify the underlying cause, cultures, fecal examination for cytology, and checking for parasitic ova are necessary. In cases of chronic enteritis in sheep and goats, ultrasonography can help assess the severity of the lesions. It can detect mild, moderate, and severe thickening of the intestinal wall, as well as the corrugation of the intestinal mucosa, both in longitudinal and cross-sectional views. Enlarged mesenteric lymph nodes are also commonly observed (51). Necropsy reveals thickened intestinal walls, corrugated mucosa in the small intestine, and enlarged mesenteric lymph nodes (Figure 8) (5).

#### 4.2.3 Retroperitoneal abscesses

A well-known form of chronic or, less frequently, intermittent colic is linked to an abscess within the abdominal cavity. These abscesses are typically retroperitoneal, and chronic leakage from them into the peritoneal cavity leads to persistent or recurrent peritonitis. Achieving full recovery is challenging, and treatment often has a high failure rate. Clinical signs indicative of this condition includes persistent or intermittent chronic colic, weight loss, and fever, along with varying levels of anorexia. In cases involving concurrent chronic peritonitis, the inflammatory exudate may accumulate enough to cause abdominal distension. Ultrasonography may help detect the abscess. Ultrasound has been reported as an effective, straightforward, and rapid diagnostic method for identifying, confirming, and planning invasive treatments for deeply located surgical lesions in sheep and goats (52).

## 5 Diseases and disorders of the liver and biliary system

In sheep and goats, the liver is made up of four distinct lobes: the right, left, quadrate, and caudate. It is located in the dorsal two-thirds of the right anterior abdomen, in contact with both the diaphragm and the abdominal wall from the seventh rib to the last rib. The liver is covered dorsally by the lung up to the ninth rib space, but biopsy access can be obtained between the ventral lung border and the costochondral junctions in the seventh to ninth rib spaces. The gallbladder extends beneath the liver’s ventral border (11).

Histologically, the central vein is positioned at the center of the hepatic lobule, and its edges are collapsed. The endothelium is visible, and the fenestrations at the margins are more pronounced near the sinusoids. The hepatic lobule is roughly hexagonal, and the hepatocytes are polygonal, with small nuclei. Hepatic sinusoids are present among the radiating cords of liver cells. In goats, a connective tissue septum exists between the portal triads but merges with the hepatic lobule. The connective tissue also contains branches of the portal vein, hepatic artery, and bile duct (13).

The gallbladder consists of several layers: the mucosa (including the epithelium and lamina propria), the muscularis, the perimuscular layer, and the serosa. The lining epithelium is tall and columnar, with a striated border. The glands are both mucous and serous, and their secretions, along with those of the surface cells, form a polysaccharide-protein complex (13).

### 5.1 Ultrasonographic examination of the hepatic parenchyma

**Figure 9:**
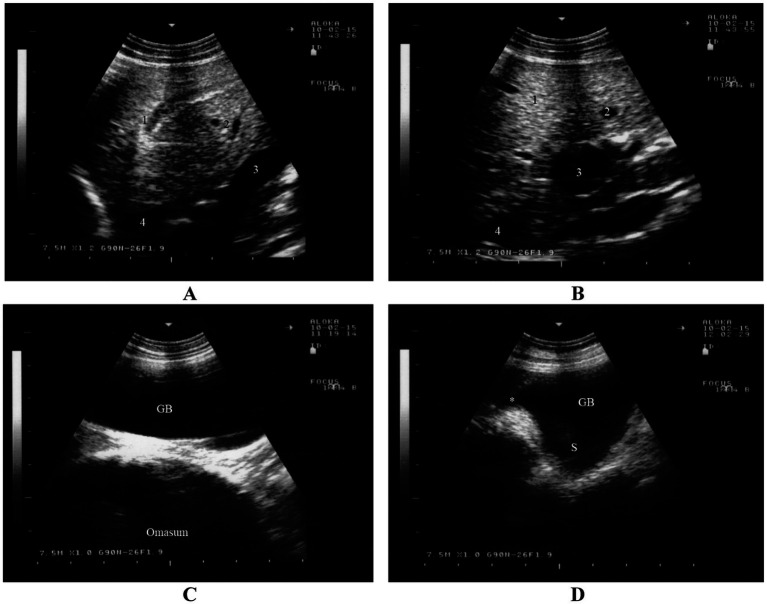
Ultrasonogram of the hepatic parenchyma in a goat. The parenchymal pattern of the normal liver consists of numerous fine echoes homogeneously distributed over the entire area of the liver. The caudal vena cava has a triangular shape on cross section because it is embedded in the sulcus of the vena cava in the liver **(A)**. The portal vein is circular to oval on cross section. The wall of the portal vein is more distinct than that of the caudal vena cava. The wall of the intrahepatic portal vein is more echoic than that of the hepatic vein **(B)**. The gallbladder is a pear-shaped and sometimes extended beyond the ventral margin of the liver depending on the amount of bile. The content of the gallbladder is anechoic to hypoechoic **(C)**. The junction of the cystic duct and the neck of the gallbladder can be imaged **(D)** (16). 1, hepatic parenchyma; 2, hepatic vein; 3, portal vein; 4, caudal vena cava. S, gallbladder sediment; *, junction of the cystic duct and the neck of the gallbladder (16).

Ultrasonographic assessment of the hepatic parenchyma is a valuable tool in clinical evaluations and plays a crucial role in diagnosing various conditions. This imaging technique offers a non-invasive, bedside approach for examining the liver parenchyma, blood vessels, as well as the renal cortex, medulla, and sinus. In sheep and goats, the dorsal margin of the liver extends in a cranioventral to caudodorsal direction, parallel to the caudal margin of the lungs. The liver’s greatest visible extent is typically seen at the seventh and eighth intercostal spaces (mean value, 15.9 cm), while its width is most noticeable at the 10th intercostal space (mean value, 5.2 cm) (11, 44). The caudal vena cava appears triangular in cross section, with its maximum width, circumference, and surface area ranging from 1.2 to 1.8 cm, 4.8 to 5.2 cm, and 0.8 to 1.1 cm^2^, respectively. The portal vein is round in cross section, with a diameter between 0.8 and 1.7 cm, and it exhibits stellate branches extending into the liver parenchyma. The gallbladder is pear-shaped and its size varies; it typically extends beyond the ventral border of the liver depending on the amount of bile present (Figure 9) (16).

Sheep and goats are often presented with nonspecific symptoms, and without hepatic ultrasonography, it can be challenging to make an antemortem diagnosis in these cases. Ultrasonographic findings almost always correlate with postmortem results, so it is recommended to routinely scan the thorax and abdomen of sheep and goats that are referred for general symptoms (6).

### 5.2 Ultrasonographic findings

Ultrasonographic examination of the liver can be performed at three or more intercostal spaces, with the liver being visible in all sheep and goats from the seventh to the ninth intercostal spaces. The dorsal margin of the liver runs parallel to the lung border, extending from cranioventral to caudodorsal (11, 44). The normal liver parenchyma exhibits a pattern of fine echoes distributed evenly throughout the organ. The caudal vena cava appears triangular in cross section, as it is located within the sulcus of the vena cava in the liver. The portal vein is round to oval in shape on cross section, and its wall is more distinct than that of the caudal vena cava. Additionally, the wall of the intrahepatic portal vein appears more echogenic compared to the hepatic vein.

The gallbladder has a pear shape and may extend beyond the liver’s ventral margin, depending on the bile volume. It can be observed at the 7th to 9th intercostal spaces, and its size varies based on the amount of bile present. The contents of the gallbladder appear anechoic to hypoechoic. Visualizing the intrahepatic bile ducts, the common hepatic duct, and the common bile duct is typically difficult. However, the junction of the cystic duct and the gallbladder neck can be imaged (11, 44).

### 5.3 Hepatic disorders

#### 5.3.1 Fatty liver

Fatty liver syndrome, also known as hepatic lipidosis, is a significant metabolic disorder with multiple contributing factors that negatively affect the health and reproductive performance of farm animals. This condition occurs when excess fat is mobilized from body fat stores to the liver, where it is deposited as triglycerides, leading to impaired liver function (40, 53, 69). Fatty liver commonly affects pregnant sheep and goats carrying multiple fetuses (pregnancy toxemia) and can also develop in the days following parturition. It is often triggered by factors that temporarily reduce the animal’s appetite, such as milk fever, indigestion, retained fetal membranes, mastitis, dystocia, or prolonged recumbency. On ultrasonography, the hepatic parenchyma appears more echogenic than usual, with the degree of echogenicity corresponding to the volume of fat vacuoles and triglyceride accumulation in the liver (53, 54). In sheep and goats and similar to dairy cows, the liver appears bright and more echogenic than healthy animals (7).

#### 5.3.2 Hepatitis cysticercosis

The disease is caused by the migration of *Cysticercus tenuicollis*, the intermediate stage of *Taenia hydatigena* (found in the intestines of dogs, coyotes, wolves, and other carnivores), into the liver and lung tissues of intermediate hosts such as sheep, goats, cattle, pigs, and squirrels (8, 55). The eggs of the parasite are excreted in feces and ingested by various domestic and wild ruminants, including goats and sheep. After hatching in the small intestine of the intermediate host, the larvae enter the bloodstream. Upon reaching the liver, they leave the portal circulation and migrate through the hepatic tissue into the peritoneal cavity, creating distinct hemorrhagic tracks within the liver.

**Figure 10:**
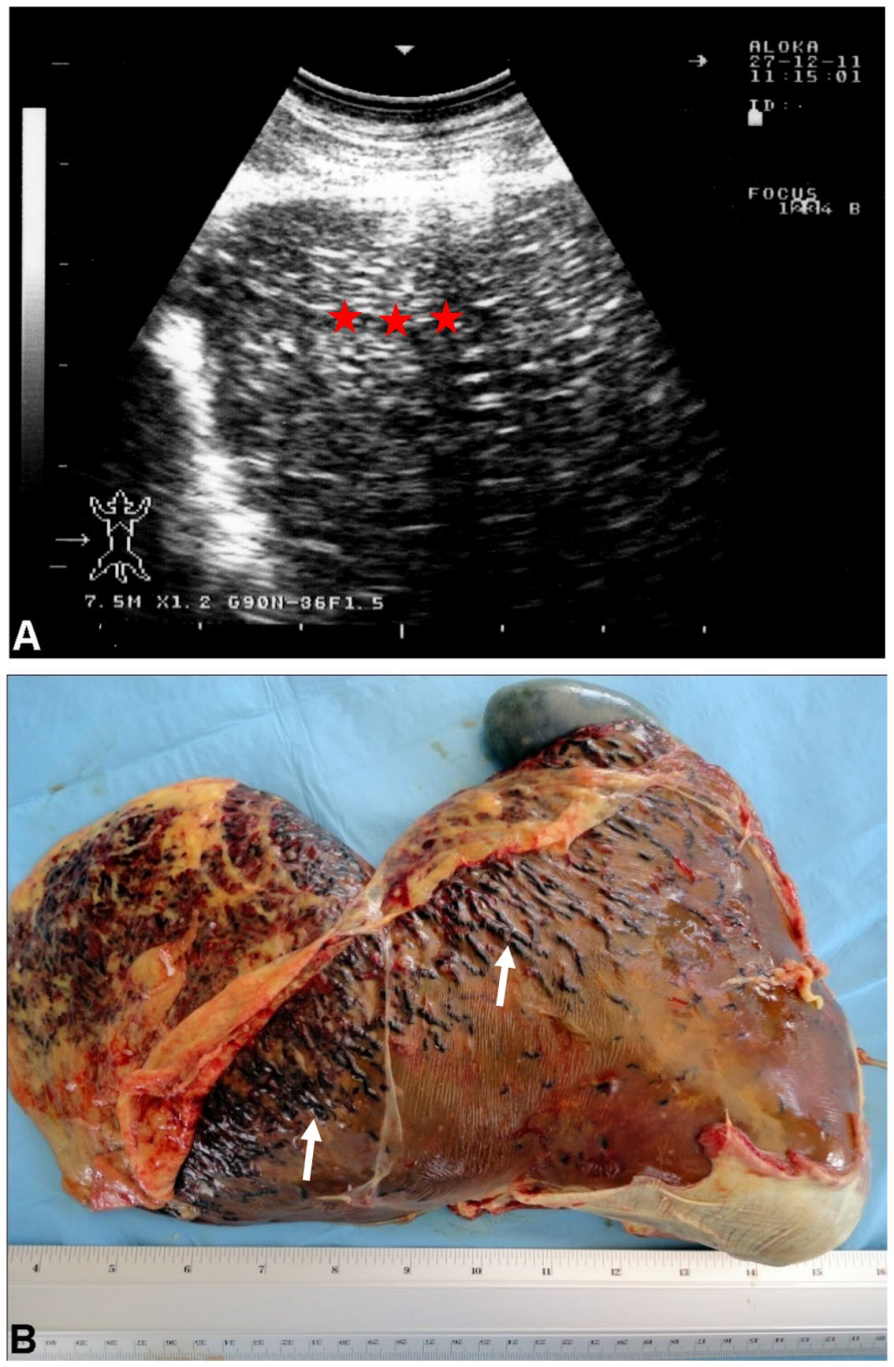
Ultrasonogram **(A)** and macroscopic view **(B)** of cysticercosis in the liver of an ovine case. **(A)** The liver parenchyma is demonstrated as heterogeneously hyperechoic (stars). **(B)** Blood-filled tracts (arrows) are seen within the swollen liver due to the migration of *Cysticercus tenuicollis* (the embryos of *Taenia hydatigena*) (58).

The metacestode, which is the stage that develops in ruminants, is known as *Cysticercus tenuicollis*. It matures over 5 to 8 weeks and attaches to the mesentery, omentum, and serosal surfaces of abdominal organs (56). In some cases, the cysticercus may remain within the liver instead of migrating, and aberrant migrations may lead to cysts being found in other organs such as the lungs (11, 20, 28, 29, 57). Peritonitis can cause fever, though chronic cysticercosis is typically asymptomatic. Ultrasonographic examination of the liver in sheep with cysticercosis reveals coarse hyperechogenic pattern (Figure 10) (8, 11, 58). At postmortem, serofibrinous or serosanguineous fluid may be present in the peritoneal cavity. Acute cysticercosis is identified by red, blood-filled tubular tracts, 2 to 4 mm in diameter, within the liver. *Cysticercus tenuicollis* cysts are commonly found in the mesentery, omentum, and serosal surface of abdominal organs, although they can also occur in the liver parenchyma. The mature cysticerci have a smooth inner surface and contain a single invaginated scolex, unlike hydatid cysts. Other postmortem findings may include migrating cysticerci in the lungs and jaundiced organs. Histopathological examination of liver tissue reveals preserved architecture with hepatitis and congestion in the large vascular spaces (8, 28).

#### 5.3.3 Cholangitis, cholecystitis, cholestasis, and choledocholithiasis

Cholangitis refers to an infection of the common bile duct, which transports bile from the liver to the gallbladder and intestines, while cholecystitis involves inflammation of the gallbladder (59). Cholestasis, on the other hand, is the cessation of bile flow. The diagnosis of obstructive cholestasis is often confirmed through the observation of a dilated bile duct and a distended gallbladder. However, it’s important to note that a distended gallbladder may also be observed at necropsy in cases of diseases that result in inappetence or anorexia (20).

In sheep and goats, common causes of cholangitis, cholecystitis, and cholestasis include fascioliasis, gallstones, and bacterial infections. Other causes of biliary tract disease include gallbladder empyema and a bile duct carcinoma (20). Ultrasonographically, thickening of the gallbladder wall is typically seen. In the differential diagnosis, it’s crucial to differentiate between gallbladder wall thickening due to right-sided heart failure, which leads to edema, and inflammatory changes confirmed at necropsy (11, 60). Choledocholithiasis, or the presence of gallstones in the common bile duct, is characterized by microliths within the duct. These stones appear on ultrasound with acoustic enhancement and an area of acoustic shadowing beneath, which can also be confirmed at necropsy (60).

Ultrasonographic findings in such cases include a dilated gallbladder with the presence of a calculus, along with dilation of the common bile duct and multiple bile ducts within the hepatic parenchyma. At postmortem, findings typically include an icteric carcass, a firm liver, and a distended gallbladder. Other necropsy results may reveal a thickened gallbladder wall, a dilated common bile duct, and a white friable mass obstructing the bile duct lumen, along with distended intrahepatic bile ducts and icteric kidneys and heart (60).

#### 5.3.4 Hydatic cyst

Cystic echinococcosis is a parasitic infection that affects various mammalian species, resulting from the larvae of *Echinococcus granulosus*, typically located in the small intestines of dogs and other carnivorous animals. Sheep, cattle, and camels serve as intermediate hosts. Liver and lung cysts caused by *E. granulosus* represent a global parasitic disease, particularly prevalent in regions where sheep are grazed with the assistance of dogs (61). The disease holds significant implications for public health at large. Humans serve as unintentional intermediate hosts (62). Sheep and goats seem to serve as the primary reservoir for human hydatiosis due to common practices such as home slaughtering, the feeding of infected offal to the definitive host and the significant prevalence of fertile cysts identified in small ruminants (63). In sonographic examinations, the intrahepatic hydatid cyst in sheep presents with indistinct borders and anechoic content that includes echogenic septa (64). The lesions can be visualized as (1) multivesicular, multiseptated cysts, indicating activity; (2) a unilocular cyst, characterized by the detachment of a laminated membrane from the cyst wall, which may harbor daughter cysts, suggesting a transitional state; and (3) a thick calcified wall that is arch-shaped, resulting in a cone-shaped shadow, indicating inactivity (19).

## 6 Diseases and disorders of the urinary system

The kidneys of sheep and goats are smooth, oval-shaped organs, with the left kidney being surrounded by more perirenal fat than the right. The right kidney is positioned in the dorsal abdomen, at the level of the T13 to L3 vertebrae, while the left kidney is situated more caudally at L4 to L5, with its lateral side in contact with the dorsal sac of the rumen. A full rumen can push the left kidney toward the right side of the abdominal midline (11). The right ureter runs alongside the vena cava, dorsal to the left kidney. The left ureter starts to the right of the midline, moves ventral to the right ureter, and then crosses back to the left to enter the bladder. The ureters pass obliquely through the bladder wall, entering the dorsal part of the bladder (28). The urinary bladder is ovoid and extends into the abdominal cavity when full, lying ventral to the uterus in females. The penis of ruminants is fibroelastic and has a sigmoid flexure. The penile urethra’s spongy part extends beyond the glans penis, forming a 2.5 cm vermiform appendage called the urethral process. In sheep, this process is attached to the left side of the glans, while in goats, it is centrally located. When the penis is flaccid, the process is folded inside the prepuce but becomes rigid and extended when erect. During ejaculation, the process rotates spirally and is thought to spray semen on the external uterine orifice or potentially enter the cervix’s external os (20).

Urinary calculi can often become trapped in the urethral process, obstructing urine flow, especially in castrated males. This is due to a reduced urethral diameter and the adhesion of the urethral process to the preputial mucosa, resulting from the loss of testosterone’s developmental effects. The urethral process is often removed during the treatment of obstructive urolithiasis to restore urine flow, and there is no evidence suggesting that this removal affects fertility in sheep and goats (29).

Histologically, the renal cortex contains renal corpuscles, tubules (including convoluted and straight parts of the nephron), collecting tubules, and an extensive vascular network. The renal corpuscle consists of a glomerular capillary bed surrounded by Bowman’s capsule. The renal medulla contains only straight tubules, ascending and descending limbs of the loop of Henle, and collecting tubules (13). The urinary bladder has three layers of smooth muscle and a multilayered transitional epithelium (urothelium). The mucosa is heavily folded to accommodate large volume changes, and the transitional epithelium can stretch to resemble stratified squamous epithelium. The submucosa consists of connective tissue, including collagen, elastin, and other extracellular matrix proteins. The bladder’s tunica muscularis is made up of the detrusor muscle, whose fibers run in all directions (13).

### 6.1 Ultrasonographic examination of the urinary system

Examination of the urinary tract is typically performed from several locations, with the most important being the flank and the last two intercostal spaces on the right side. These sites allow visualization of the right kidney and, in most goats, the left kidney as well. The urinary bladder and urethra can be assessed transrectally, but can also be seen from either the left or right inguinal region. The same equipment used for examining other organs is employed for transcutaneous ultrasound, while transrectal exams are best conducted with a 5.0 to 7.5-MHz probe (44, 65).

The right kidney is generally visible from the right, with the optimal viewing site being the dorsal region of the 11th or 12th intercostal space and from the craniodorsal flank. The right kidney is almost always oriented with its long axis parallel to the ribs, and only rarely does it appear perpendicular to them. The left kidney can typically be seen in most sheep and goats from the right, and less often from the left, though in some cases, it may not be visible from either side. When seen from the right, the left kidney is most commonly viewed from the dorsal flank or occasionally from the last intercostal space. On the left side, the left kidney may be observed, though rarely, from the dorsal flank. The long axis of the left kidney is usually parallel to the vertebral column (3, 11, 44).

**Figure 11:**
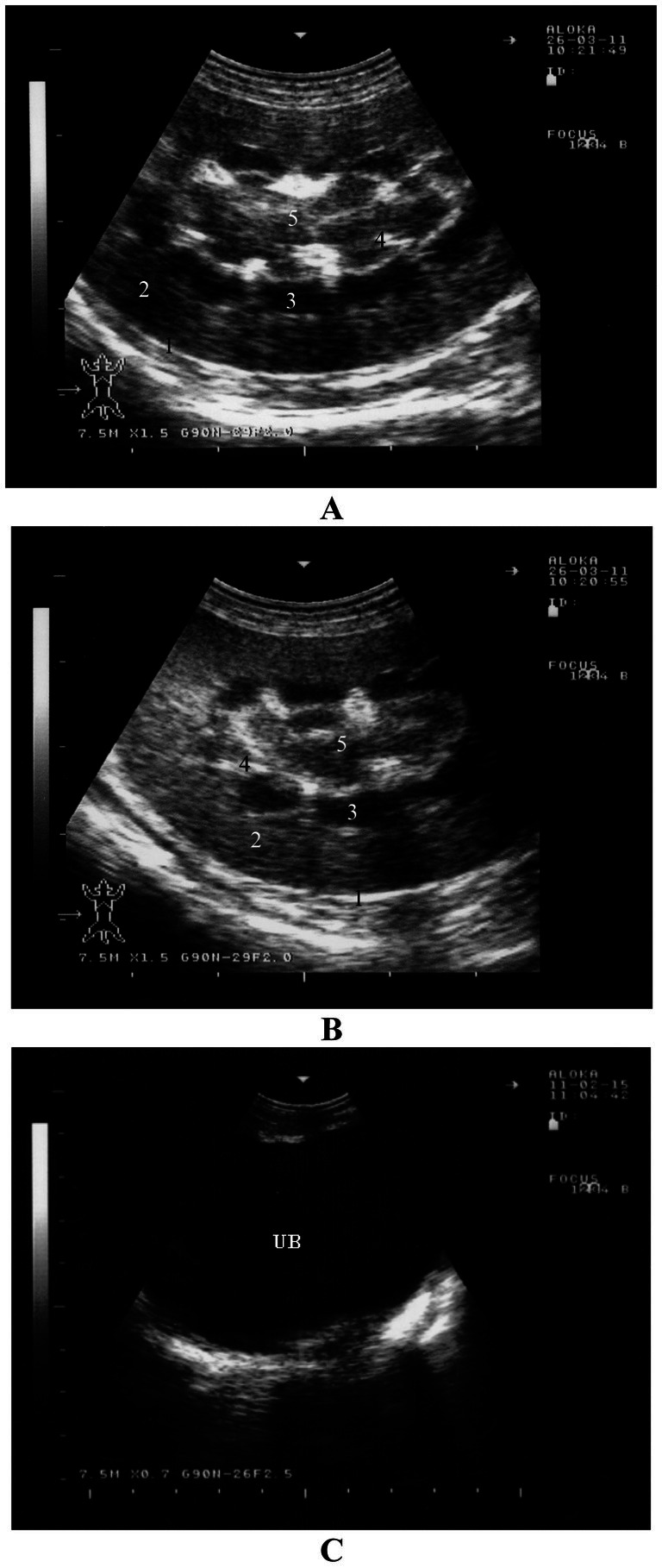
Ultrasonography of the kidneys and urinary bladder in a healthy goat. Image **(A,B)** show ultrasonograms of the right **(A)** and left **(B)** kidneys in longitudinal section through the region of the medullary pyramides viewed from the right flank. Image **(C)** shows transrectal imaging of the urinary bladder (UB) with anechoic urine 1, renal capsule; 2, renal cortex; 3, medullary pyramids; 4, interlobar vessels; 5, renal sinus (16).

The renal capsule may appear as a faint echoic line, though it is not always clearly visible. The kidneys’ ultrasonographic appearance changes depending on the sectional plane. In a sagittal plane through the hilus, the kidney has an oval shape, and its parenchyma appears homogeneous with fine, evenly distributed echoes. The medullary pyramids near the sinus appear as oval to circular hypoechoic structures, and the sinus itself appears hyperechoic. In a longitudinal section through the medullary pyramids, the kidney remains oval, with the renal cortex being distinct and easily differentiated from the renal medulla and pyramids. The interlobar veins and arteries appear as elongated hypoechoic structures between the pyramids, and the renal sinus is visible as a hyperechoic band. Oblong hypoechoic structures within the renal sinus correspond to the renal artery, vein, and ureter (Figures 11A,B) (16). Non-dilated ureters are typically not visible through either the flank or transrectally (65). The urinary bladder is most clearly visible during a transrectal examination, although it can also be seen from either inguinal region. The bladder wall appears as an echoic line with smooth borders, and its contents are normally anechoic. In most sheep and goats, the urethra is seen transrectally as two parallel echoic lines, but the lumen is usually not visible (Figure 11C) (16).

### 6.2 Urinary disorders

#### 6.2.1 Renal failure

Renal function is influenced by the number and proper functioning of the nephrons. Renal failure can be categorized into pre-renal, renal, and post-renal causes (20, 66). Common clinical signs of renal failure include depression, loss of appetite, weight loss, oral mucosal lesions, and diarrhea. A male goat presented to our clinic exhibited the typical clinical signs of renal failure. The animal showed signs of depression, lethargy, and loss of appetite, with abdominal distension being the first noticeable symptom. Abdominocentesis revealed a significant amount of watery abdominal fluid. Ultrasonography showed increased echogenicity of the renal cortex, the presence of a kidney calculus in the right kidney, and an effusion of anechoic fluid in the abdomen. Blood tests indicated elevated serum levels of blood urea nitrogen (64.3 mmol/L, with a normal range of 3–10 mmol/L) and creatinine (1,637 μmol/L, with a normal range of 70–105 μmol/L).

#### 6.2.2 Hydronephrosis

**Figure 12:**
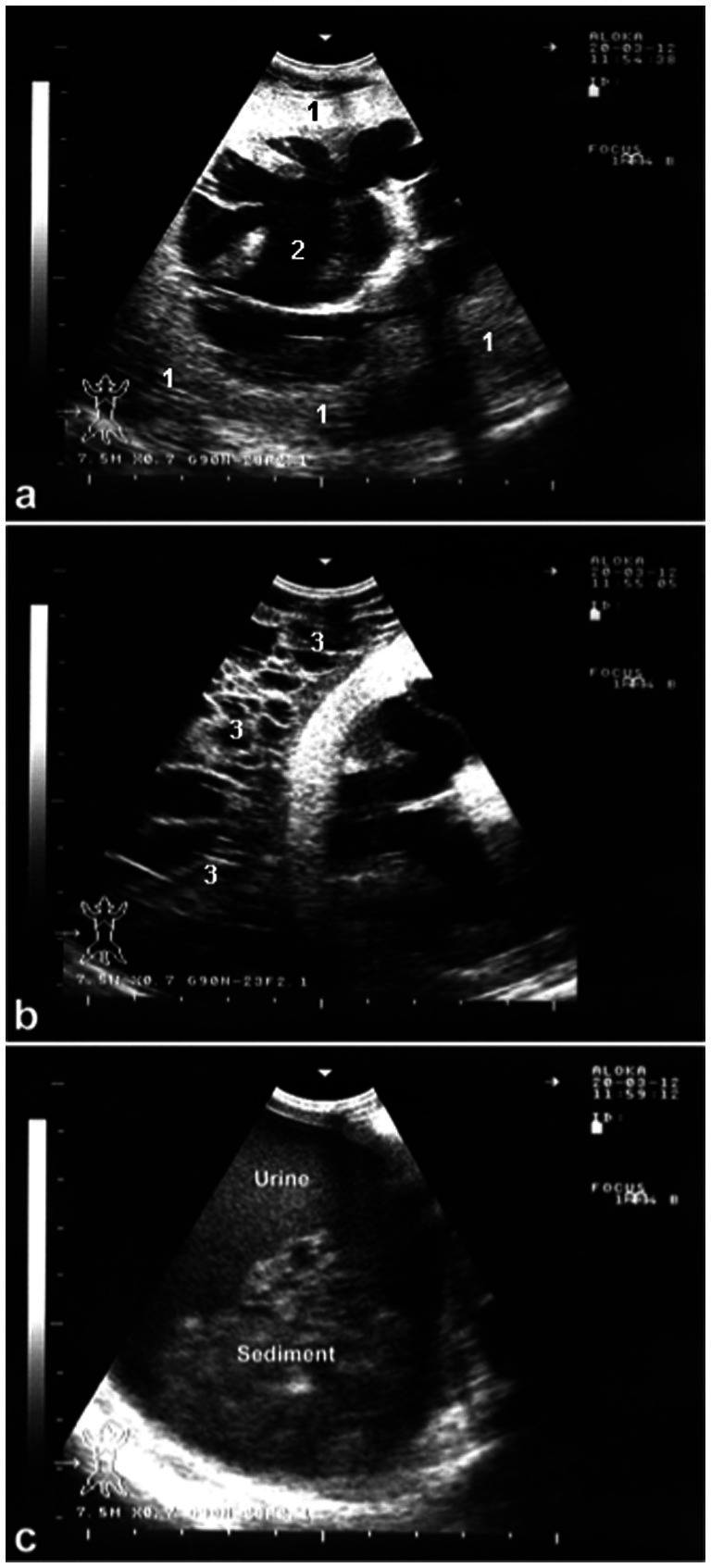
Ultrasonographic findings in a ram with hydronephrosis using a 7.5 MHz convex transducer: **(A)** Severely enlarged kidney (1) with dilated renal pelvis (2) and pressure atrophy of the renal parenchyma; **(B)** perirenal edema (3); **(C)** ventral sludge in the urinary bladder (3).

Hydronephrosis is the enlargement of the renal pelvis accompanied by progressive atrophy of the renal parenchyma. It can be either congenital or acquired due to urinary tract obstruction. Any blockage in the urinary tract may lead to hydronephrosis, with the severity of the renal damage depending on the extent and duration of the obstruction (66). Chronic, unilateral, and partial urinary tract obstructions are more likely to cause hydronephrosis. In cases of acute and complete obstruction, symptoms typically include anuria, dysuria, or stranguria. Chronic or partial obstructions result in gradual dilation of the renal pelvis and pressure-induced atrophy of the renal tissue (29). When the obstruction is unilateral, the unaffected kidney may compensate for the loss of function, potentially preventing kidney failure. Ultrasonography can help in diagnosing hydronephrosis, revealing a dilated renal pelvis and signs of pressure atrophy in the renal parenchyma (Figure 12). Postmortem examination can confirm whether the condition is unilateral or bilateral.

#### 6.2.3 Pyelonephritis

**Figure 13:**
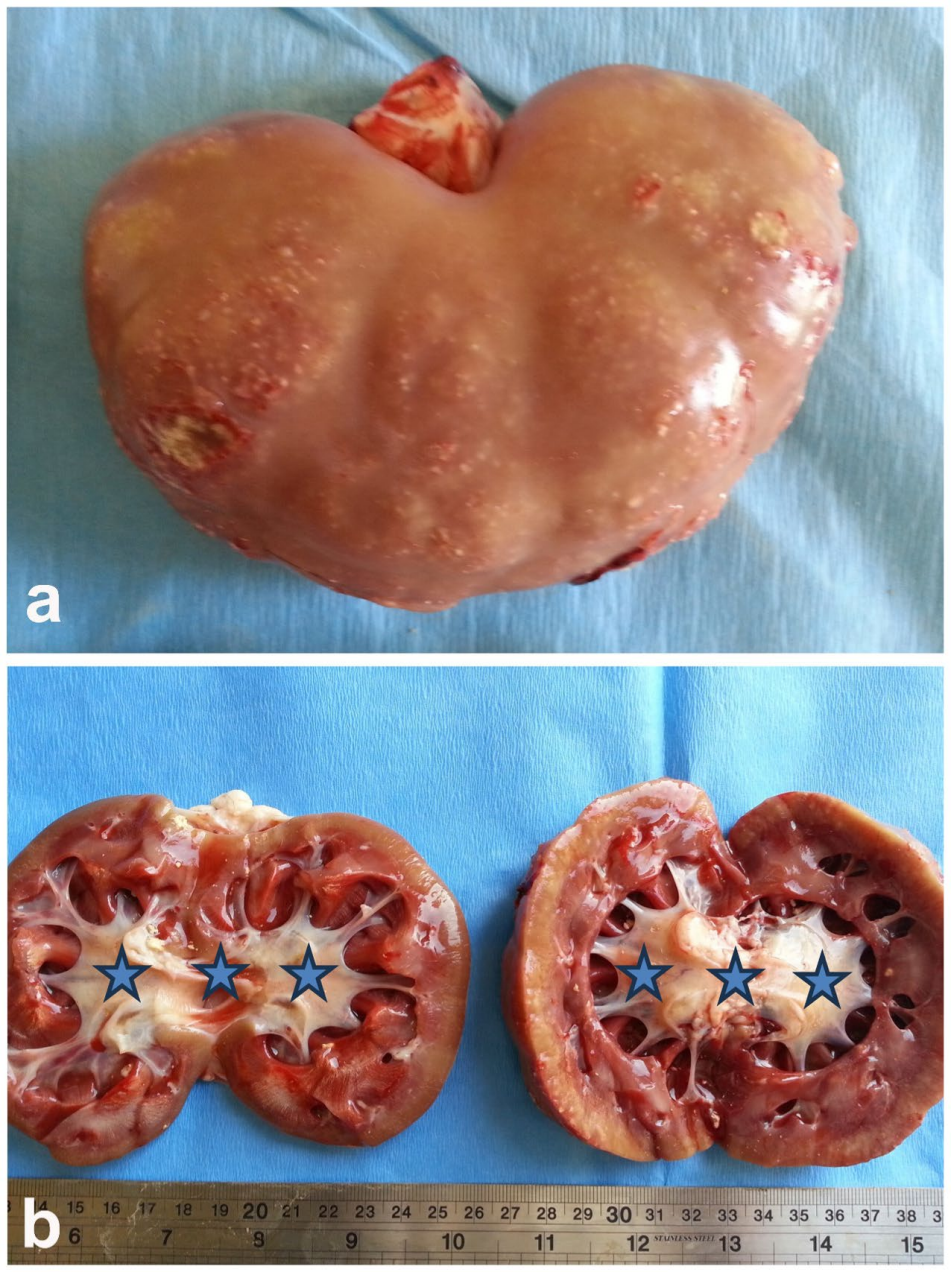
Postmortem finding of a buck with pyelonephritis. **(A)** Sub-capsular pinpoint micro-abscesses. **(B)** Bilateral dilatation of the renal pelvises (stars) (3).

Pyelonephritis is an uncommon condition in sheep and goats, and it can be caused by organisms other than *Corynebacterium renale*, such as *Trueperella pyogenes* (previously *Arcanobacterium pyogenes*). The relatively low rate of post-partum uterine infections in sheep and goats, compared to cattle, may partly explain why this ascending kidney infection is less frequent in these animals. The disease generally progresses slowly and is characterized by symptoms such as fever, colic, pyuria, hematuria, cystitis, ureteritis, and suppurative nephritis. Ultrasonography can assist in diagnosing pyelonephritis by showing irregularly shaped kidneys with a loss of the corticomedullary boundary, as well as hypo- or hyperechoic changes in the renal cortex and increased echogenicity. Additionally, hyperechogenic urine and bladder sediment containing cellular debris, red blood cells, and pus cells may be detected (9). Upon postmortem examination, the affected kidney is often enlarged, with lesions in various stages of development within the parenchyma with dilated renal pelvis (Figure 13). Necrotic gray streaks may extend from the medulla to the cortex, and the kidney may show multicystic changes. Histological examination reveals widespread suppurative pyelonephritis in both the renal cortex and medulla (11, 28).

#### 6.2.4 Obstructive urolithiasis

Obstructive urolithiasis refers to the inability to pass urine due to blockage in the urinary outflow tract caused by calculi. In sheep and goats, this condition is most commonly seen in young, castrated males, with the calculi typically composed of phosphate salts, such as calcium phosphate (apatite) and magnesium ammonium phosphate (struvite). The formation of these urinary stones is influenced by various physiological, nutritional, and management factors. The likelihood of urinary calculi getting stuck in the urethra is linked to anatomical features and the practice of castrating male ruminants (10, 66). Ultrasonography of the urinary system may reveal the presence of calculi, a collapsed bladder, and uroperitoneum. Ruptured urethra should be distinguished from abdominal herniation. In an adult goat buck that was admitted with a ruptured urinary bladder, abdominocentesis showed reddish urine, with red blood cell sediment found after centrifugation. The urethral process and glans penis were congested and blackish. Ultrasonography indicated a distended bladder with uroperitoneum. At necropsy, dissection of the urinary tract revealed calculi, urethral trauma, and rupture of the bladder or urethra. Calculi were found in the renal cortex, renal pelvis, or within the urethral process. Other postmortem findings included a collapsed and perforated bladder, bilateral hydronephrosis, and uroperitoneum (11, 20, 28, 29).

#### 6.2.5 Cystitis

**Figure 14:**
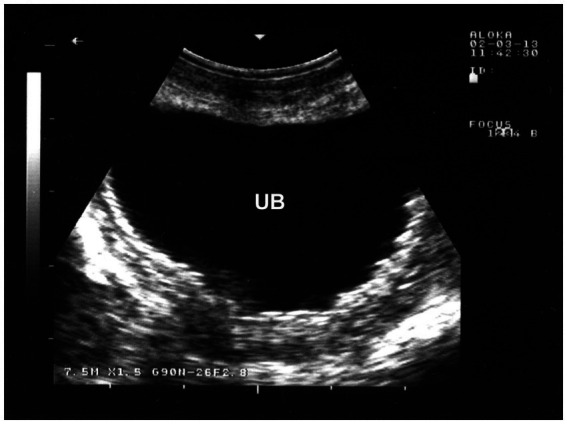
Ultrasonographic findings in a goat with cystitis showing the thickened and corrugated mucosa of the urinary bladder (UB). Image was taking using a 7.5 MHz convex transducer (3).

Inflammation of the urinary bladder typically occurs as a result of bladder paralysis, which leads to urine retention, dystocia, ascending infection from the urethra, or chronic irritation caused by cystic calculi (28). Clinically, the condition is marked by frequent, painful urination (pollakiuria and dysuria), as well as the presence of blood (hematuria), inflammatory cells, and bacteria in the urine. The urine may visibly contain blood or pus. A microscopic urinalysis may show pyuria and the presence of bacteria. Ultrasonographic imaging of the inflamed bladder typically reveals a thickened, corrugated mucosal lining (Figure 14) (66).

#### 6.2.6 Paralysis of the urinary bladder

**Figure 15:**
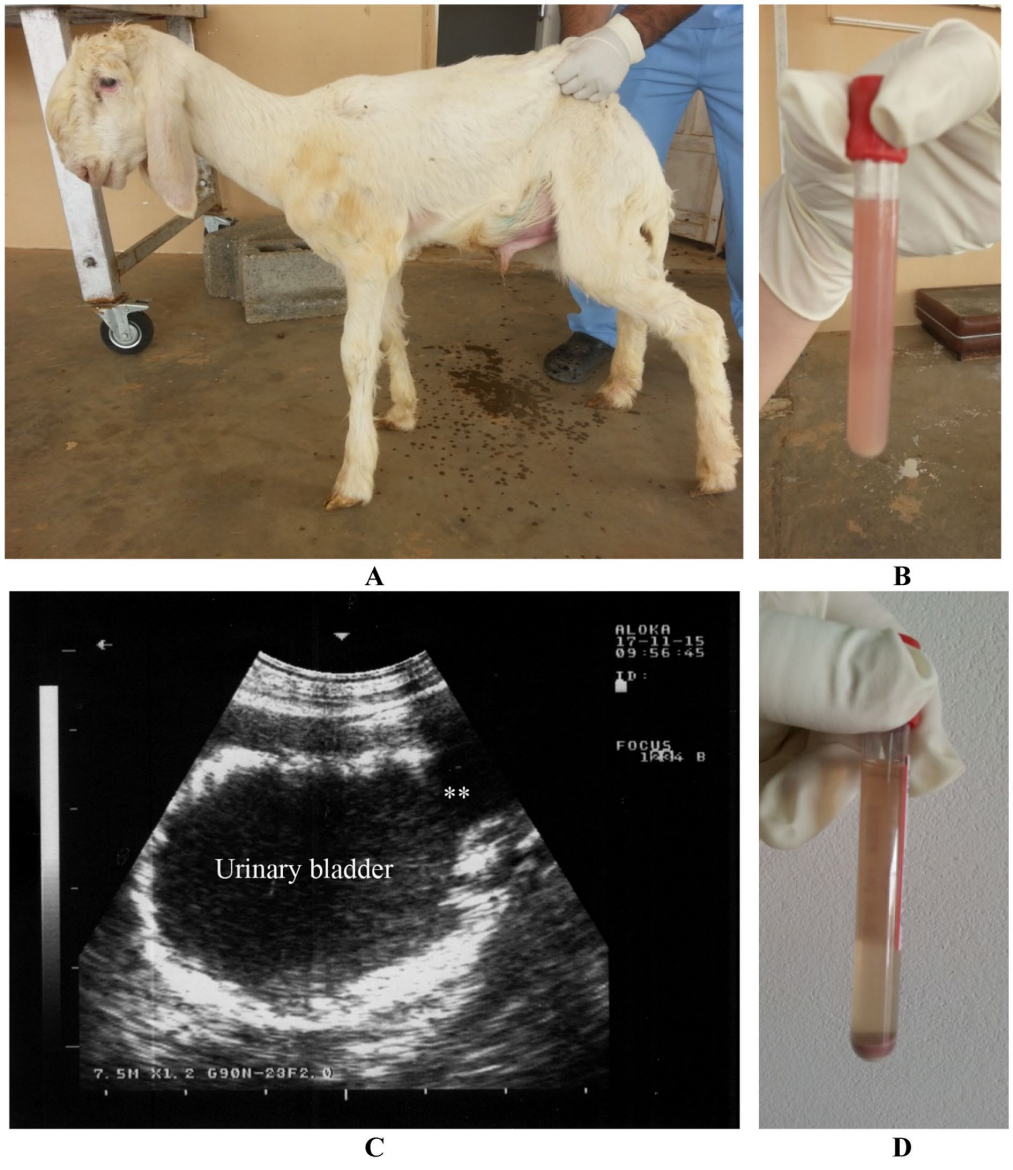
Paralysis of the urinary bladder in a lamb. Incontinence with dribbling of urine was the owner’s complaint **(A)**. Urine sample was cloudy **(B)**. Ultrasonographic examination reveals dilated bladder, thickened wall, echogenic content and dilated bladder neck (stars) **(C)**. Urine sediment is clear upon centrifugation **(D)** (3).

Bladder paralysis is rare in large animals and typically results from neurological conditions that affect the lumbosacral spinal cord. In lambs, ascending spinal meningitis is a significant cause, especially following tail docking. In all species, bladder paralysis can also occur due to compression of the lumbar spinal cord by tumors (such as lymphosarcoma or melanoma) or infected tissue (such as vertebral osteomyelitis) (20). Incontinence, with constant or intermittent urine dribbling, often worsens during physical activity. Abdominal examination reveals an enlarged bladder, and urine can be easily expelled through manual compression. The retention of urine creates favorable conditions for bacterial growth, often leading to cystitis. Ultrasonography shows a dilated bladder with a thickened wall, echogenic contents, and a dilated bladder neck. Urine sediment remains clear after centrifugation of a sample (11) (Figure 15).

## 7 Conclusion

This review article highlights the effectiveness of sonography in both healthy and diseased sheep and goats. The technique can be employed to assess the normal structure and function of healthy thoracic and abdominal organs. Additionally, it serves as a valuable tool for diagnosing and predicting respiratory, cardiovascular, gastrointestinal, hepatic, and urinary conditions. It is strongly recommended to use ultrasonography as an initial diagnostic tool when evaluating sheep and goats with thoracic and abdominal medical issues. While ultrasonography is a valuable tool for examining sheep and goats, there are few limitations to its use such as operator skill, technical equipment limitations, animal stress and limited depth penetration especially in well-fattened animals. These limitations highlight the need for careful consideration when choosing ultrasonography as a diagnostic tool for sheep and goats.
